# A Web-Based Self-Help Intervention With and Without Chat Counseling to Reduce Cannabis Use in Problematic Cannabis Users: Three-Arm Randomized Controlled Trial

**DOI:** 10.2196/jmir.4860

**Published:** 2015-10-13

**Authors:** Michael P Schaub, Andreas Wenger, Oliver Berg, Thilo Beck, Lars Stark, Eveline Buehler, Severin Haug

**Affiliations:** ^1^ Swiss Research Institute for Public Health and Addiction (ISGF) associated to the University of Zurich and World Health Organization Collaborating Center Zurich Switzerland; ^2^ Arud Center for Addiction Medicine Zurich Switzerland

**Keywords:** cannabis, Internet, chat, Web based, self-help, cognitive behavioral therapy, motivational interviewing, counseling, self-control, behavioral self-management

## Abstract

**Background:**

After alcohol and tobacco, cannabis is the most widely used psychoactive substance in many countries worldwide. Although approximately one in ten users develops serious problems of dependency, only a minority attend outpatient addiction counseling centers. A Web-based intervention could potentially reach those users who hesitate to approach such treatment centers.

**Objective:**

To test the efficacy of a Web-based self-help intervention with and without chat counseling—Can Reduce—in reducing the cannabis use of problematic cannabis users as an alternative to outpatient treatment services.

**Methods:**

Altogether, 436 participants were recruited by various online and offline media for the Web-based trial. A total of 308 of these were eligible for study participation and were randomly allocated in an unblinded manner to either self-help with chat (n=114), self-help without chat (n=101), or a waiting list control group (n=93). The fully automated self-help intervention consisted of eight modules designed to reduce cannabis use, and was based on the principles of motivational interviewing, self-control practices, and methods of cognitive behavioral therapy. Additional individual chat counseling sessions were based on the same therapeutic principles. The sessions were conducted by trained counselors and addressed participants' personal problems. The main outcomes were the frequency (number of days) and quantity of cannabis use (number of standardized joints) per week, as entered into the consumption diary at baseline and at the 3-month follow-up. Secondary outcomes included self-reported symptoms of cannabis use disorder, severity of cannabis dependence, risky alcohol use, and mental health symptoms. Intervention participation and retention were extracted from the user progress data and the consumption diary, respectively.

**Results:**

Can Reduce participants were older (U=2.296, *P*=.02) and reported a greater number of cannabis use days at baseline than patients who entered outpatient treatment with cannabis as their main problem substance (data from the Swiss treatment demand monitoring statistics were used; chi-square [df 2]=4.0, *P*=.046). Participants in the self-help with chat study arm completed a mean of 3.2 modules and 27 out of 114 (23.7%) of the participants received at least one chat session. Participants in the self-help without chat study arm completed similar numbers of self-help modules. A total of 117 of 308 participants (38.0%) completed the 3-month follow-up assessment. The change in the mean number of cannabis use days per week at 3 months differed between self-help without chat (mean change 0.7, SD -0.2) and self-help with chat (mean change 1.4, SD -0.5; beta=-0.75, SE=0.32, t=-2.39, *P*=.02, d=0.34, 95% CI 0.07-0.61), as well as between self-help with chat and waiting list (mean change 1.0, SD -0.8; beta=0.70, SE=0.32, t=2.16, *P*=.03, d=0.20, 95% CI -0.07 to 0.47). However, there were no differences between self-help without chat and waiting list (beta=-0.05, SE=0.33, t=-0.16, *P*=.87, d=-0.14, 95% CI -0.43 to 0.14). Self-reported abstinence was significantly different in the self-help without chat study arm (2.0%) than in the self-help with chat study arm (8.8%; beta=-1.56, SE=0.79, *P*=.05, odds ratio [OR]=0.21, 95% CI 0.02-2.33). There were no significant differences between the study arms with respect to the secondary outcomes.

**Conclusions:**

Web-based self-help interventions supplemented by brief chat counseling are an effective alternative to face-to-face treatment and can reach a group of cannabis users who differ in their use and sociodemographic characteristics from those who enter outpatient addiction treatment.

**Trial Registration:**

International Standard Randomized Controlled Trial Number (ISRCTN): 59948178; http://www.isrctn.com/ISRCTN59948178 (Archived by WebCite at http://www.webcitation.org/6bt01gfIr)

## Introduction

Web-based self-help programs that aim to reduce cannabis use might help to reach cannabis users who do not want to enter available outpatient addiction counseling services due to their fear of being stigmatized or their need to distance themselves socially from drug counselors [1]. Moreover, the limited opening hours of many outpatient addiction services might act as a barrier to care for some users [1]. It has been estimated that approximately 22% of Europeans between 15 and 64 years of age have tried cannabis. A total of 6.8% of Europeans report using cannabis in the preceding month and an estimated 3 million report daily cannabis use [2]. Switzerland has the third-highest national prevalence of cannabis use in Europe; the 12-month prevalence rate is 5.7% (men 7.8%, women 3.7%) and the 30-day prevalence rate is 2.7% (men 3.7%, women 1.7%) [3]. The age group with the highest prevalence is between 15 and 24 years of age; this group has a 12-month prevalence rate of 19.9%, and nearly one in five members from this group uses cannabis daily [3]. Daily cannabis use is associated with greater risks of developing cannabis dependence, poor mental and physical health, lower educational achievement, and decreased cognitive functioning [4]. The risks of cannabis dependence [5] and problems with cannabis use [6] are considerably higher in cannabis users with early rather than late onset of use.

Treatment demand statistics from Swiss in- and outpatient addiction treatment centers demonstrated a linear increase—from 2006 (9.9%) to 2012 (14.7%)—in new treatment entry cases for whom cannabis was the main problem substance [7]. The main group seeking treatment for cannabis use disorder mainly consists of adolescents and young adults between the ages of 15 and 24 years old (71.6%) and are predominantly male (82.5%) [8]. In Europe, cannabis is the main problem substance for almost 40% of all individuals entering addiction treatment for the first time and has been a more frequent problem than opioids since 2006 [9]. It has been estimated that about 50% of problematic cannabis users will develop cannabis dependence [5] and many of these exhibit mental health problems; however, most of them are not yet in treatment. Raising awareness of cannabis-related risks to physical health might also encourage users to reduce or quit cannabis use [10]. In general, the principle of stepped care (ie, noninvasive, low-cost interventions in which therapeutic intensity can be enhanced according to need) appears to be an appropriate means for problematic cannabis users to lower their ever-increasing health care costs [11], and this consideration is of interest in Switzerland and other industrialized countries suffering from exorbitant health costs.

An initial meta-analysis included diverse studies that mainly investigated computer- and some Web-based interventions to reduce cannabis consumption and found a small overall effect size (g=0.16, 95% CI 0.09-0.22, *P*<.001) at posttreatment. There have now been three studies on the efficacy of Web-based interventions to reduce cannabis use in problematic users. First, the German *Quit the Shit* program [12] is based on principles of self-regulation and self-control and is a solution-focused approach. This program is structured into weekly personalized feedback sessions based on participants’ consumption diary entries, and intake and termination chats; the total allowed program time is 50 days. Tossmann et al [12] recruited a total of 1292 cannabis users and found significant reductions in cannabis use in their intention-to-treat (ITT) analyses, but with high attrition rates. Second, a distinct version of the program was developed that consisted of one comprehensive chat session with motivational interviewing (MI) [13] in the intervention group (n=33) versus a technical information chat in the control group (n=34). No significant differences in cannabis use were found between the study groups [14]. Third, the Australian program, *Reduce Your Use: How to Break the Cannabis Habit* [15], is a fully automated self-help intervention consisting of six modules that aim to reduce the symptoms of cannabis use disorders and which is based on cognitive behavioral therapy (CBT) [16,17], MI [13], and behavioral self-management (BSM) [18]. Its efficacy was tested in a randomized controlled trial (RCT) and compared to a psychoeducative control condition that also consisted of six modules (n=225). The frequency of cannabis use and the quantity of cannabis consumed were both reduced to a greater extent in the intervention group than in the control group at 6 weeks and at the 3-month follow-up. They achieved considerably higher participation rates at the 3-month follow-up than the German *Quit the Shit* program (54% in the intervention and 52% in the control condition) [12].

The combination of a fully automated self-help intervention based on the approaches of Rooke et al [15], together with additional individual chat sessions to reduce cannabis use, could potentially increase the efficacy of interventions for problematic cannabis users—in the sense that the use is harmful to the user or others—as has been demonstrated for the reduction of alcohol use in problematic alcohol users [19].

Thus, the current study aims to investigate and compare the efficacy of Web-based self-help interventions—in combination with or without tailored chat counseling based on CBT, MI, and BSM—in reducing cannabis use in problematic cannabis users.

## Methods

### Participants

Study participants were recruited by a press release, several websites from local outpatient treatment centers, and from nightlife prevention websites that were linked to the Can Reduce website [20]. In addition, advertisements were placed in Internet forums and recruitment flyers were distributed to Swiss addiction service centers and practitioners in the Canton of Zurich. Moreover, two major Swiss commuter newspapers and one Swiss weekend newspaper published extensive reports on the Can Reduce interventions in their print media and websites. The collaboration of the Swiss Research Institute for Public Health and Addiction (ISGF) and the Arud Centers for Addiction Medicine (ARUD) as the responsible study institutions was clearly stated in all recruitment channels.

Study inclusion and exclusion criteria are depicted in Table 1. In addition to the email addresses in the registration process, participants were asked to provide their telephone numbers in case they could not be reached online for the 3-month follow-up [1]. The participant information and informed consent page from the Can Reduce website is provided in Multimedia Appendix 1.

**Table 1:** Inclusion and exclusion criteria and rationales.

Participant criteria		Rationales
*Inclusion criteria*		
	Minimum age of 18 years	To ensure a minimal age of participation
	Read and understand German	To ensure understanding of interventions
	Internet access and a valid email address	To ensure participation
	Using cannabis at least once a week over the 30 days prior to study entry	To include at least occasional users
*Exclusion criteria*		
	Current serious psychiatric disorders or history of psychosis, schizophrenia, bipolar type I disorder, or significant current suicidal or homicidal thoughts	To avoid exacerbation of serious symptoms of these severe psychiatric disorders
	Other pharmacological or psychosocial treatments for cannabis use disorders	To avoid confounding treatment effects
	For women: pregnancy and breastfeeding	To avoid serious complications resulting, for example, from withdrawal symptoms

### Preparatory Work

The Web-based self-help intervention, Can Reduce, was based on classical CBT approaches for treating cannabis dependence [17], MI approaches [13], and BSM [18]. A detailed description of the intervention can be found in the study protocol by Schaub et al [1]. This randomized controlled trial was registered with the International Standard Randomized Controlled Trial Number (ISRCTN) registry (ISRCTN59948178).

Can Reduce is the first self-help intervention for problematic cannabis users in Switzerland. It was developed by the authors of this publication from the ISGF and the ARUD. Both institutions are located in the Canton of Zurich, Switzerland. Study participation was free of charge. The self-help part of Can Reduce was developed according to the experiences of an earlier study in problematic cocaine users [21,22] and the Global Drug Survey cannabis meter [23], and was piloted for acceptability and usability. The piloting was organized into two steps. In the first step, we piloted Can Reduce with cannabis-using students from the University of Zurich. In the second step, we combined this with additional chat sessions with two trained psychiatrists from ARUD and four of their problematic cannabis-using patients. This pilot phase resulted in some minor changes in the interventions.

### Ethical Review and Informed Consent

The protocol of the RCT was approved by the Ethics Committee of the Canton of Zurich (KEK-StV-Nr. 15/13) and was carried out in compliance with the Helsinki Declaration. Before giving informed consent, participants were informed of the following: (1) the rationale of the study, (2) study inclusion and exclusion criteria (see Table 1), (3) the three different arms and their 1:3 chance of being allocated to one of the arms, (4) the potential risks of participation, (5) safety arrangements during and after the study phase [19], (6) the inability of Can Reduce (with or without chat counseling) to replace face-to-face therapy for problematic cannabis use/abuse, (7) the circumstances under which they should contact their general practitioner or a professional from a medical advisory group; an emergency list that would be accessible at all times via an instant help button was provided as well, (8) the approval of the study by the Ethics Committee of the Canton of Zurich and their declaration of no objection (nihil obstat), and (9) their right to withdraw from the study at any time without consequences except for the loss of further compensation. Informed consent was accepted when participants clicked on all consent fields of the informed consent page and submitted the consent by clicking the submission button (see Multimedia Appendix 1).

### Study Arms and Contents

There were three different study arms. The first consisted of the Web-based self-help intervention, Can Reduce, in combination with up to two individual chat counseling sessions based on MI and CBT approaches that considered the data the participants entered into the self-help intervention and individual requests. The second study arm consisted of the same intervention but without chat counseling. Study arms 1 and 2 received weekly automated motivational emails to remind the user to log in and fill out the consumption diary. Study arm 3 consisted of a classical waiting list and people in this arm received access to the self-help intervention after 3 months.

The following modules, organized into three main parts, were offered as a Web-based self-help intervention (study arms 1 and 2) and—as long as the participant did not feel an urgent need to skip to a specific module—it was recommended that they should be worked through in the order shown in Textbox 1 within the planned 6 weeks of intervention.

**Modules for the Can Reduce Web-based self-help intervention.**

Part 1: Introduction

**Figure 1:**
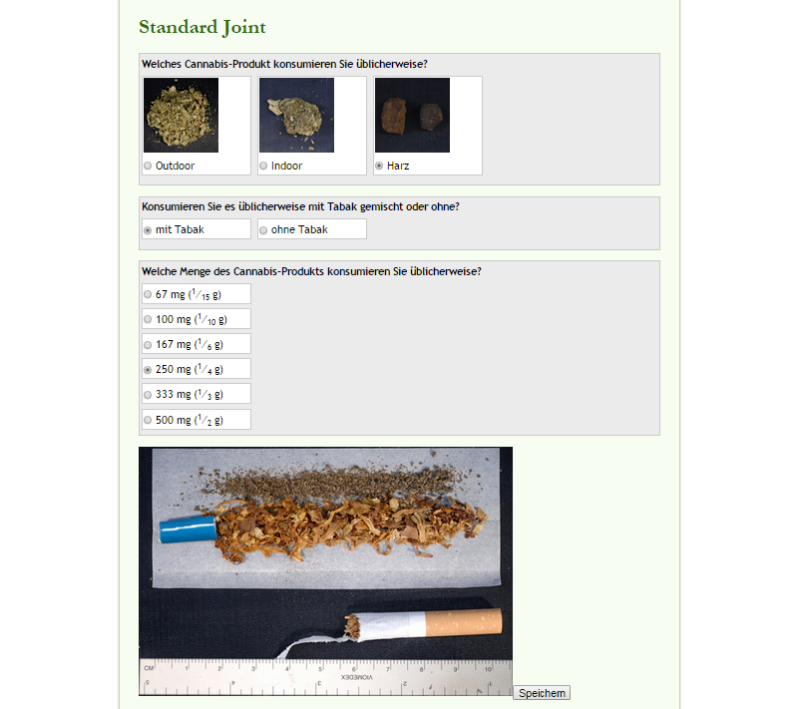
Screenshot of the Can Reduce Web-based intervention, showing the decision on the standard cannabis joint prior to the first consumption diary entry.

- Registration process
- Explanation of the "standard cannabis joint" concept and choice of the personal standard cannabis joint (see Figure 1), the cannabis consumption diary, and the automated reminder emails
- Examination of the pros and cons resulting from a change in cannabis consumption patterns and further principles of motivational interviewing to address motivation, followed by setting an appropriate target value for overall cannabis use, which is to be reached by the end of the intervention
- Explanation of the *My Can Reduce* folder
- Explanation of the emergency button for immediate responses to frequently asked questions and access to emergency contacts

**Figure 2:**
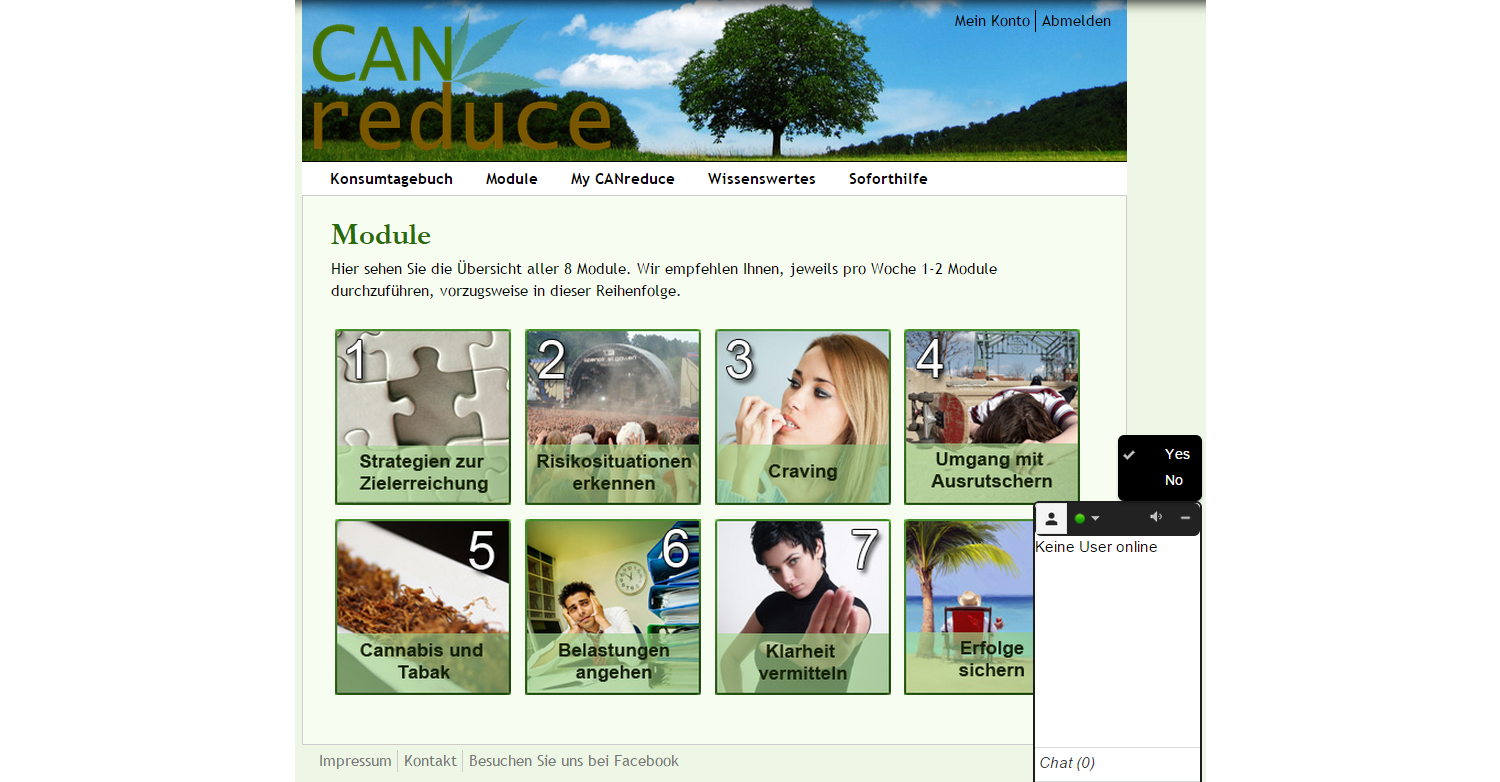
Main menu of the Can Reduce Web-based intervention's study arm 1 with self-help plus chat counseling that took place within the website in a small box at the bottom right corner.

Part 2: Key Modules (participants are encouraged to complete these modules in the order presented below; see Figure 2)

- Module 1: Strategies for goal achievement
- Module 2: Identifying risk situations
- Module 3: Dealing with cannabis craving
- Module 4: Dealing with relapses

Part 3: Further Modules (participants are encouraged to complete at least two, in any order)

- Module 5: Tobacco smoking during the reduction in cannabis use
- Module 6: Saying "no" to foster refusal skills
- Module 7: Dealing with burdens
- Module 8: Preserving achievements

Figures 1 and 2 show screenshots of the Can Reduce Web-based intervention website. The following were also provided: a glossary that explained the terms, definitions, and concepts used in the intervention; a knowledge base about the history of cannabis use; the effects and risks of cannabis use; concurrent mental health problems; and the enhanced risks when cannabis is mixed with tobacco and smoked, as in a previously developed and positively evaluated cannabis group smoking cessation program [10,24] (study arm 1 and 2). The knowledge base also included harm reduction techniques with recommendations for the use of cannabis [25,26].

The additional (up to two) chat counseling sessions with a scheduled duration of 20 to 30 minutes in study arm 1 supported behavioral change according to MI, discussed the modules of the Web-based self-help part based on MI and CBT, and reviewed the development of the consumption diary. Invitations to chat sessions were sent by the counselors according to a predefined procedure between weeks 1 and 2 for the first and between weeks 4 and 6 for the second chat session. The chats took place within the website in a small box at the bottom right corner, while keeping the content of the webpage in view (see Figure 2). It was initially planned that the structure of these chat sessions should be fixed [1]. However, as a result of the counselor supervision sessions, the structure of the chat session was made more flexible and more dependent on the participants' needs and served as a checklist for the counselors in order to ensure that they covered all of the relevant contents.

The chat counselors received quarterly supervision sessions and consisted of trained MI counselors, mainly psychologists or psychiatrists with advanced or completed further education, with at least one year of experience in treating cannabis-abusing patients face to face. Specific quality standards were developed for addiction chat counseling and implemented for this study in the chat counselor supervision based on the study on the development of a European Union framework for minimum quality standards and benchmarks in drug demand reduction treatment quality standards [27] and the Swiss national addiction counseling quality standards [28].

To optimize and manage their interactions with clients, counselors had access to a specific user management area to add arranged chat dates, define statuses, and add personal comments about their clients. With this tool, counselors could follow their clients’ progress in reducing their cannabis use through clearly arranged charts, and look up previous chat histories. Specific lists helped counselors track their clients (eg, a list with *all users*, *my clients*, or *my upcoming chat sessions*).

The Web-based self-help intervention and the subsequent tailored chat counseling aimed to reduce cannabis use. However, those participants who sought cannabis abstinence were also encouraged to make step-by-step reductions until full abstinence was reached. In accordance with the counselor supervision group, we deviated from the study protocol [1] by introducing the option to dispense with a second chat session if a participant and his/her counselor agreed that another chat would not be needed.

Participants randomized to the waiting list had the opportunity to participate in the Web-based self-help intervention 3 months after registration.

### Detailed Study Hypotheses

This study aimed at comparing the efficacy of a Web-based self-help intervention alone or combined with chat counseling in the reduction of the cannabis use of problematic cannabis users within a three-arm randomized controlled trial with assessments at baseline and 3-month follow-up (see Multimedia Appendix 2 for the CONSORT-EHEALTH checklist [29]).

We hypothesized that Web-based interventions—which are more interactive—would be more effective than less interactive interventions in reducing cannabis use among problematic cannabis users. We tested the following detailed study hypotheses with respect to the main outcome (ie, the reduction of the weekly cannabis used between the baseline and the 3-month follow-up):

1. Tailored chat-based counseling in combination with Web-based self-help for the reduction of cannabis use (study arm 1) is more effective than the waiting list control condition (study arm 3).
2. Web-based self-help for the reduction of cannabis use (study arm 2) is more effective than the waiting list control condition (study arm 3).
3. Chat-based counseling in addition to Web-based self-help for the reduction of cannabis use (study arm 1) exhibits a trend to be more effective than Web-based self-help alone (study arm 2).

### Measurement Instruments

The primary outcome measure was the recorded quantity of cannabis use in the previous 7 days, quantified in individually standardized cannabis joint sizes, and as specified in the consumption diary (see Table 2 and Schaub et al for further details [1]). In the first step, participants chose between three different cannabis forms presented in photographs—low-potency cannabis plant, high-potency cannabis plant, or cannabis resin (see Figure 1). In the second step, five different standard joints for each category were presented (1/10 g, 1/6 g, 1/4 g, 1/3 g, 1/2 g; pictures came from the Global Drug Survey cannabis meter [23]); these joints were either pure cannabis or cannabis mixed with tobacco. A standard tobacco cigarette, a ruler with centimeter and millimeter scales, the fraction amount in grams, and an open 10 cm paper prepared to roll a joint and containing the cannabis plant-/resin-tobacco mixture or pure cannabis were presented. Participants chose which picture most closely approximated the cannabis joints they most often smoke. The chosen picture was placed in the individual consumption diary (see Figure 1), and participants were asked to convert the quantities of cannabis they smoked into units relative to that picture if they exceptionally consumed cannabis in forms other than their common standard joint. As this kind of outcome assessment has not previously been used in an efficacy trial, we also considered the number of cannabis use days in the last 7 days as a primary outcome [1].

The following secondary outcome instruments were applied:

1. The Cannabis Use Disorders Identification Test (CUDIT), which is a 10-item questionnaire [30] that was constructed by adapting the Alcohol Use Disorders Identification Test [31]. To cover the length of the trial, this instrument was adapted to focus on the last 3 months in its planned assessments (baseline and 3-month follow-up).

2. The Severity of Dependence Scale (SDS), which is a five-item questionnaire that measures the severity of cannabis dependence. Each of the five items is scored on a 4-point scale (0-3). The total score is obtained by adding the ratings on all five items. High scores indicate high levels of dependency [32].

3. The Cannabis Withdrawal Scale (CWS) [33], which is a 19-item questionnaire containing statements that describe cannabis withdrawal symptoms within the last 24 hours on an 11-point scale (0-10).

4. The Cannabis Craving Symptoms questionnaire (CCS-7), which is a seven-item questionnaire [34] derived from the Marijuana Craving Questionnaire [35]. Each item is rated on a 7-point scale (1-7).

5. The Fragebogen Substanzanamnese (FDA), which is a questionnaire that ascertains the number of years of consumption over the lifetime, the past month’s consumption, and the manner of consumption for the Diagnostic and Statistical Manual of Mental Disorders’ substances of abuse. This measure was derived from the Europe Addiction Severity Index [36].

6. The short version of the Mental Health Inventory (MHI-5) [37], which is a validated and user-friendly self-assessment questionnaire that assesses recent mental distress and self-reported diagnoses of depression.

None of the secondary outcome instruments has yet been specifically validated for Internet use. Intervention satisfaction for all modules, the diary, the chat, the knowledge base, the instant help, and the overall satisfaction was ascertained on a 4-point scale, ranging from *not at all useful* to *very useful*. Finally, intervention participation was assessed for completed modules each time a participant pressed the *back to the main menu* button at the very end of a module. Retention was calculated as the percentage of days per week a user entered any number of cannabis use in the diary.

**Table 2:** Study measurements and instruments.

Assessments/instruments	Baseline	1 week	3 weeks	6 weeks	3-month follow-up
Sociodemographics	x				
MHI-5^a^	x				x
Quantity of cannabis use^b^	x	x	x	x	x
Frequency of cannabis use^b^	x	x	x	x	x
CUDIT^c^	x				x
SDS^d^	x			x	x
FDA^e^	x			x	x

^a^Mental Health Inventory (MHI-5).

^b^7-day point prevalence values of the quantity (in common standard joints) and frequency (the number of days on which cannabis is used) of cannabis use were derived from the consumption diary for the preceding 7 days.

^c^Cannabis Use Disorders Identification Test (CUDIT).

^d^Severity of Dependence Scale (SDS).

^e^Fragebogen Substanzanamnese (FDA).

### Sample Size

Based on results of the study of Rooke et al [15], we expected small to medium effect sizes of at least 0.30 (Cohen’s d) for the reduction in the quantity of cannabis used and the frequency of cannabis use between study arm 2 (Web-based self-help without chat counseling) and study arm 3 (waiting list control) between baseline and follow-up assessment, and greater effects between study arms 1 and 3. We estimated a sample size of 89 in each study group that would have 80% power (*F* test, alpha = 5%) to detect these differences, as based on calculations with G*Power software version 3.1. Therefore, we aimed to recruit a total of 267 participants [1]. We had no reference values for the expected differences in effects between study arms 1 and 2 and thus planned an exploratory study of effect sizes in case we failed to reach significance for these study arm comparisons.

### Randomization and Allocation

Once participants had completed their baseline assessment, they were randomized by a computer program in a 1:1:1 ratio to one of three parallel groups. As the participant information offered full transparency on the three study arms in our nonblinded design, we anticipated a risk that some participants might register another account, in an effort to change their assignment and access a different study arm. In that case, the participant remained in the initially assigned study arm for the rest of the day, as based on his or her IP address.

### Statistical Methods

Data were analyzed according to the intention-to-treat principle. For the ITT analyses, in departure from the study protocol, we applied multiple imputation procedures of R (R Foundation for Statistical Computing, Vienna, Austria) in Amelia II that have been demonstrated to outperform other imputation methods [38]. For each study arm, we performed 50 separate imputations using the following as imputation variables: sex, age, education, origin, years of cannabis use, number of finished modules, the baseline variables for frequency and quantity of cannabis use, alcohol use in the last 30 days (risky and normal), SDS, CUDIT, and MHI-5. Baseline measurements were compared between the three study arms and study participants were compared with people entering addiction treatment according to data from the Swiss *addiction, care and therapy information* (act-info) monitoring statistics. Depending on the scale of the corresponding outcome, Mann-Whitney U tests, chi-square tests, or analyses of variance (ANOVA) were calculated via SPSS version 22.0 (IBM Corporation). The calculation of the changes between baseline and the 3-month follow-up was modified from the protocol, as there were a considerable number of missing values at the 6-week assessment. Regression analyses in R were used for the calculated differences between 3-month follow-up and baseline, using the corresponding baseline variables as control variables. Results from the imputed dataset were cross-checked with the nonimputed dataset in the latter analyses. In departure from the study protocol, we dispensed with analyzing the Cannabis Withdrawal Scale and the Cannabis Craving Symptoms questionnaire data [1], as very low numbers of questionnaires were completed at intervention weeks 3 and 6. In the study dropout analysis, we conducted regression analyses to investigate the interaction effect of relevant baseline characteristics (ie, sociodemographic and consumption characteristics) between those who did and those who did not provide a 3-month follow-up. These analyses were conducted for the total sample and for each study arm separately. Similar analyses were conducted in the subgroup analyses.

## Results

### Participant Flow

**Figure 3:**
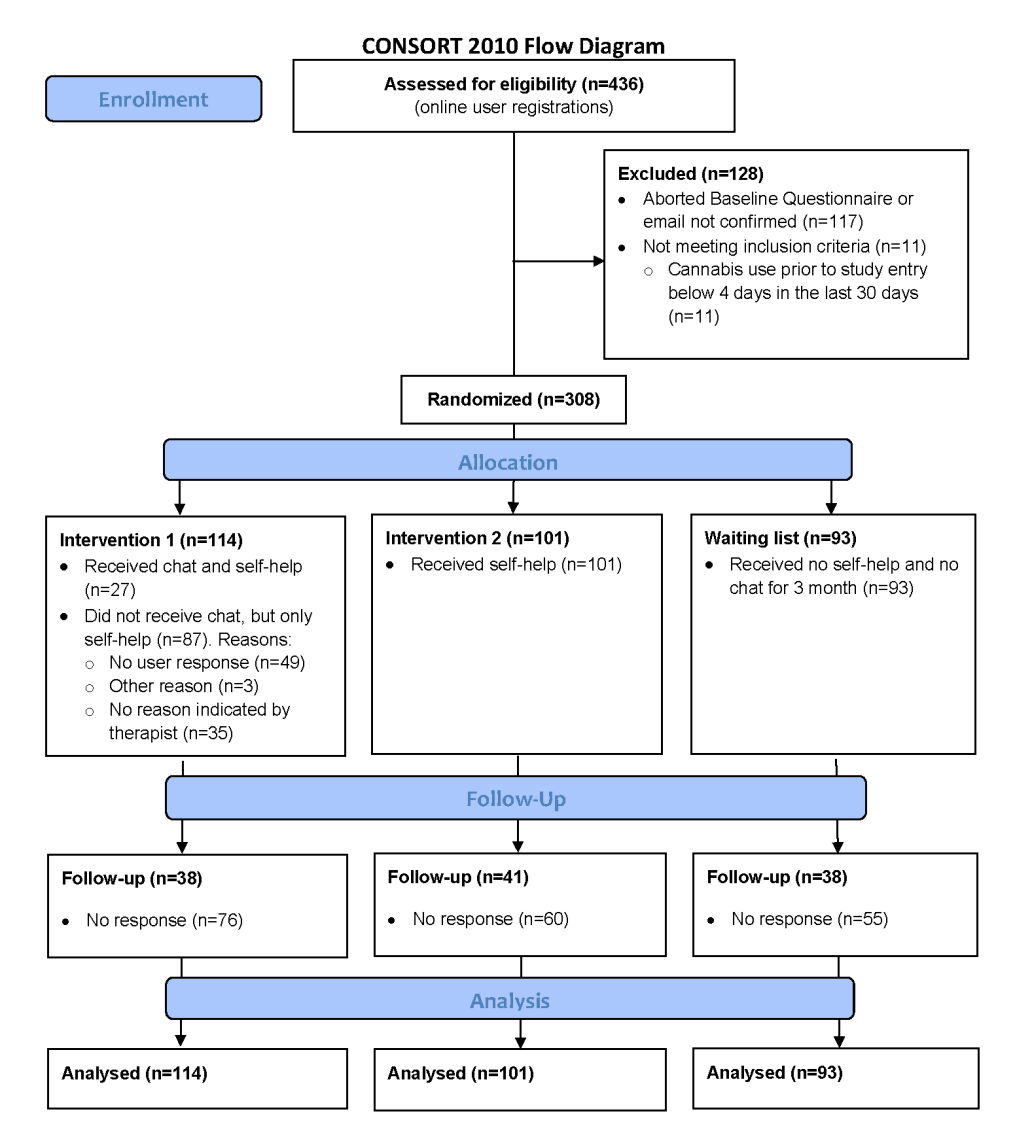
CONSORT-EHEALTH trial flowchart: overview of the participant flow for this trial.

Figure 3 provides an overview of the trial flow. Recruitment started in the beginning of June 2014 and ended on February 28, 2015, after exceeding the total estimated number of 267 participants. Of the 436 Can Reduce registrants recruited, 308 (70.6%) were allocated to one of the three study arms.

Three months after the baseline assessment, participants were invited by email to log in and complete the final study assessment; they were reimbursed with €40 (via an online voucher or an online charitable donation). The follow-up assessment was performed in three steps. First, participants were invited via email to participate in the assessment. Up to three reminders were sent. Those participants who failed to complete the 3-month follow-up despite these reminders were contacted via telephone and offered an interview by study collaborators. Those participants who refused a telephone interview were offered an interview on the primary outcome only. Finally, 117 out of 308 participants (38.0%) could be followed up with.

### Participants’ Baseline Characteristics

Table 3 provides an overview of the participants’ characteristics and comparisons between the three study arms at baseline assessment. In comparison with participants whose main problem substance was cannabis in the Swiss treatment monitoring statistics (act-info) in 2013 [7], Can Reduce participants demonstrated a similar gender distribution (75.3% males in Can Reduce vs 82.4% act-info, U=1.342, *P*=.18), tended to be older within the age groups between 20 and 69 years old (U=2.296, *P*=.02), and reported a higher number of cannabis use days in the 7 days prior to intervention start (70.9% daily use in Can Reduce vs 41.4% act-info; 20.2% 4-6 days per week Can Reduce vs 10.5% act-info; 5.2% 2-3 days per week Can Reduce vs 21.7% act-info; 3.8% 1 day a week Can Reduce vs 26.3% act-info; chi-square [df 2]=4.0, *P*=.046).

**Table 3:** Baseline characteristics of participants.

Characteristics		Study arm 1^a^ (n=114)	Study arm 2^b^ (n=101)	Study arm 3^c^ (n=93)	Total (n=308)	χ^2^, ANOVA^d^, or Kruskal-Wallis test	*P*
**Sex, n (%)**						χ^2^_2_=4.3 (n=308)	.12
	Female	35 (30.7)	24 (23.8)	17 (18)	76 (24.7)		
	Male	79 (69.3)	77 (76.2)	76 (82)	232 (75.3)		
Age in years, mean (SD)		28.4 (9.6)	30.2 (9.2)	31.0 (11.1)	29.8 (10.0)	*F*_2,308_=1.940	.15
**Age range, n (%)**						χ^2^_2_=3.9 (n=308)	.14
	≤20 years	24 (21.1)	12 (11.9)	18 (19)	54 (17.5)		
	21-25 years	31 (27.2)	19 (18.8)	13 (14)	63 (20.5)		
	26-30 years	16 (14.0)	29 (28.7)	19 (20)	64 (20.8)		
	31-35 years	17 (14.9)	18 (17.8)	15 (16)	50 (16.2)		
	36-40 years	14 (12.3)	10 (9.9)	11 (12)	35 (11.4)		
	41-45 years	6 (5.3)	5 (5.0)	7 (8)	18 (5.8)		
	46+ years	6 (5.3)	8 (7.9)	10 (11)	24 (7.8)		
**Highest education, n (%)**						χ^2^_10_=8.6 (n=308)	.57
	Not specified	4 (3.5)	3 (3.0)	5 (5)	12 (3.9)		
	Primary school	18 (15.8)	12 (11.9)	11 (12)	41 (13.3)		
	Apprenticeship	43 (37.7)	38 (37.6)	41 (44)	122 (39.6)		
	Secondary school	19 (16.7)	13 (12.9)	17 (18)	49 (15.9)		
	Technical college	18 (15.8)	26 (25.7)	13 (14)	57 (18.5)		
	University	12 (10.5)	9 (8.9)	6 (7)	27 (8.8)		
**Origin, n (%)**						χ^2^_6_=8.1 (n=308)	.23
	Canton of Zurich	52 (45.6)	33 (32.7)	42 (45)	127 (41.2)		
	Other cantons	53 (46.5)	61 (60.4)	48 (52)	162 (52.6)		
	Germany	8 (7.0)	5 (5.0)	3 (3)	16 (5.2)		
	Other countries	1 (0.9)	2 (2.0)	0 (0)	3 (1.0)		
CUDIT^e^, mean (SD)		19.8 (5.8)	19.7 (6.4)	19.1 (6.2)	19.6 (6.1)	*F*_2,308_=0.37	.69
SDS^f^, mean (SD)		7.7 (3.5)	7.5 (3.6)	7.3 (3.2)	7.5 (3.4)	*F*_2,308_=0.37	.69
MHI-5^g^, mean (SD)		54.0 (19.3)	53.9 (20.0)	55.1 (22.6)	54.3 (20.5)	*F*_2,308_=0.11	.90
**Number of years of substance use, mean (SD)**							
	Cannabinoids	9.6 (7.4)	10.9 (7.6)	12.6 (10.0)	10.9 (8.4)	*F*_2,305_=3.29	.04^h^
	Risky alcohol use^i^	2.5 (5.6)	2.6 (5.3)	2.7 (6.4)	2.6 (5.7)	*F*_2,228_=0.03	.97
	Cocaine	1.1 (4.3)	1.4 (3.8)	0.8 (1.8)	1.1 (3.4)	*F*_2,222_=0.67	.52
	Amphetamines	0.7 (2.0)	1.1 (3.1)	0.6 (2.0)	0.8 (2.4)	*F*_2,208_=0.91	.40
**Substance use in the last 30 days, n (%)**							
	Cannabinoids	112 (98.2)	100 (99.0)	93 (100)	305 (99.0)	Not computable (no variance)	N/A^j^
	Risky alcohol use^i^	40 (35.1)	26 (25.7)	31 (33)	97 (31.5)	χ^2^_2_=2.6 (n=226)	.28
	Tranquilizers	7 (6.1)	8 (7.9)	5 (5)	20 (6.5)	χ^2^_2_=0.6 (n=215)	.74
	Cocaine	7 (6.1)	14 (13.9)	10 (11)	31 (10.1)	χ^2^_2_=2.7 (n=223)	.26
	Amphetamines	16 (14.0)	13 (12.9)	14 (15)	43 (14.0)	χ^2^_2_=0.2 (n=221)	.90
	Hallucinogens	6 (5.3)	4 (4.0)	4 (4)	14 (4.5)	χ^2^_2_=0.6 (n=210)	.76
	Heroin	0 (0)	1 (1.0)	0 (0)	1 (0.3)	χ^2^_2_=1.8 (n=201)	.40
	Methadone	1 (0.9)	1 (1.0)	3 (3)	5 (1.6)	χ^2^_2_=1.9 (n=197)	.40
	Others	4 (3.5)	1 (1.0)	1 (1)	6 (1.9)	χ^2^_2_=3.1 (n=198)	.21

^a^Self-help with chat.

^b^Self-help without chat.

^c^Waiting list.

^d^Analysis of variance (ANOVA).

^e^Cannabis Use Disorders Identification Test (CUDIT) scores range from 0 to 40 with a cutoff of >8 for a cannabis use disorder.

^f^Severity of Dependence Scale (SDS) scores range from 0 to 15 with a cutoff of ≥4 for cannabis dependence.

^g^Mental Health Inventory (MHI-5): higher values represent improved symptoms. MHI-5 values range from 0 to 100 with a cutoff of <70 for clinically relevant symptoms.

^h^ *P*<.05, represents a significant value.

^i^Risky alcohol use was defined as five or more standard drinks per day on at least three days per week. A standard drink was defined as 5 cl spirits, 15-20 cl wine, or 33-45 cl beer.

^j^Not applicable (N/A).

### Intervention Participation and Retention

**Figure 4:**
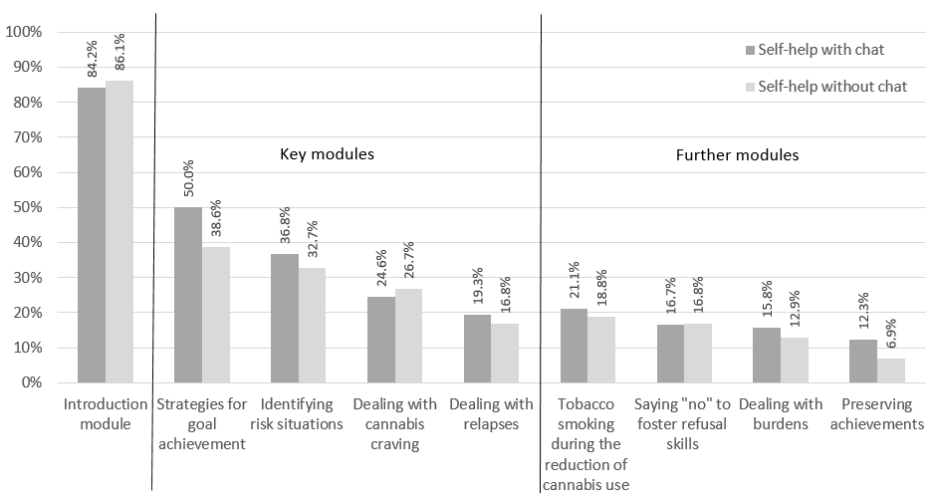
Module completion rate for study arms 1 (self-help with chat) and 2 (self-help without chat).

**Figure 5:**
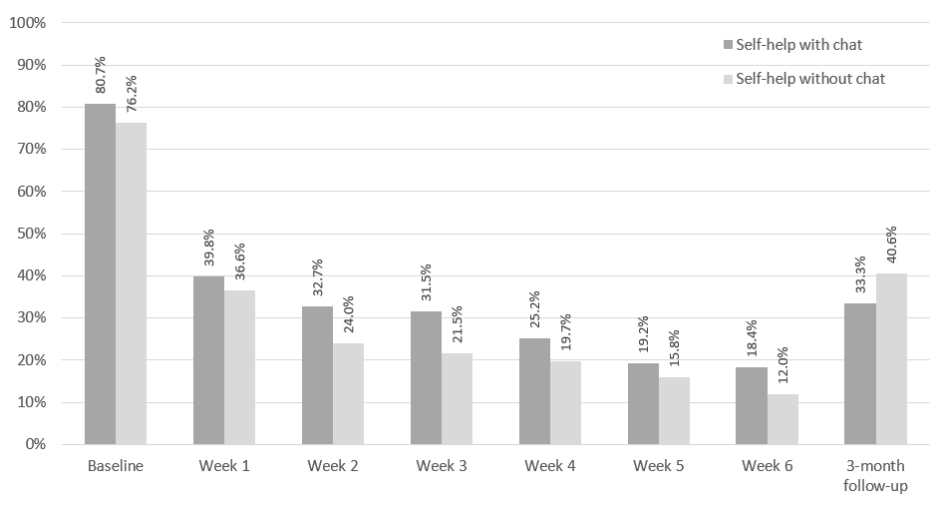
Study retention based on the weekly completion of the consumption diary for study arms 1 (self-help with chat) and 2 (self-help without chat) between baseline and week 6, including 3-month follow-up completion rate.

Figure 4 depicts the module completion by participants in study arms 1 and 2. Participants in the self-help with chat study arm completed a mean of 3.2 modules and 27 out of 114 (23.7%) of the participants received at least one chat session. Participants in the self-help without chat study arm completed similar numbers of self-help modules (U=-1.189, *P*=.23). Participants in study arm 1 more frequently completed the consumption diary than those in study arm 2 during their recommended 6 intervention weeks (U=-2.375, *P*=.02; see Figure 5). Of the 27 users in study arm 1 who received chat counseling sessions, 23 (85%) received one session and 4 (15%) received two sessions.

### Main Outcomes

**Figure 6:**
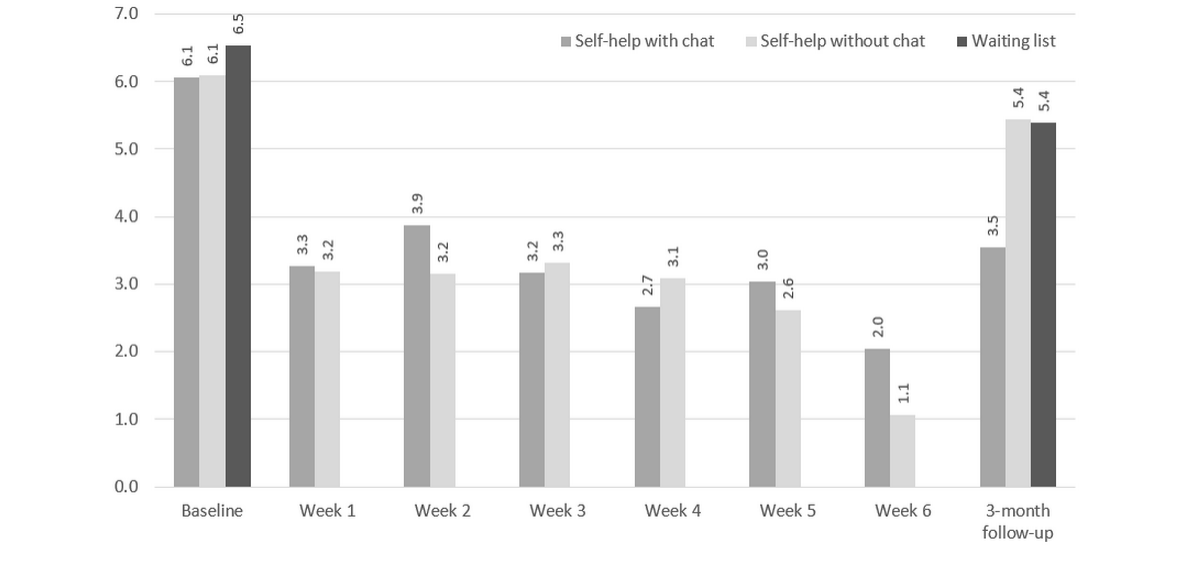
Cannabis use days per week according to the consumption diary between baseline and 3-month follow-up for all three study arms based on the nonimputed dataset.

**Figure 7:**
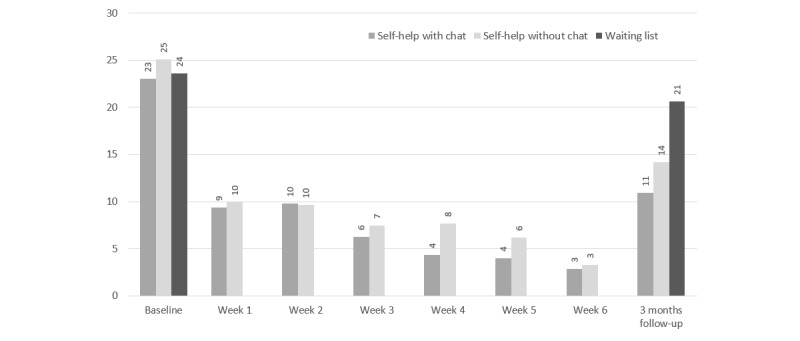
Weekly quantity of cannabis used in number of standardized cannabis joints between baseline and 3-month follow-up for all three study arms based on the nonimputed dataset.

Figure 6 depicts the mean numbers of cannabis use days per week and Figure 7 the mean weekly quantity of cannabis used in standard joints according to the consumption diary, between baseline and follow-up for all three study arms and based on the nonimputed dataset.

The differences in cannabis use between baseline and the 3-month follow-up, as expressed by the mean number of cannabis use days per week and based on the imputed data, differed between self-help without chat versus self-help with chat (beta= -0.75, SE = 0.32, *t*=-2.39, *P*=.02, d=0.34, 95% CI 0.07-0.61), and between self-help with chat versus waiting list (beta= 0.70, SE = 0.32, *t*=2.16, *P*=.03, d=0.20, 95% CI -0.07 to 0.47), but not between self-help without chat versus waiting list (beta= -0.05, SE = 0.33, *t*=-0.16, *P*=.87, d=-0.14, 95% CI -0.43 to 0.14). In contrast, we only observed one trend to a significant difference in the weekly quantity of standard joints in the comparison of self-help with chat versus waiting list in the imputed dataset (beta = 4.73, SE = 2.50, *t*=1.89, *P*=.06, d=0.09, 95% CI -0.19 to 0.36; see Tables 4 and 5).

### Secondary Outcomes

There were no significant differences in the group comparisons in the secondary outcomes (see Tables 4 and 5). We observed slight improvements in mental health (MHI-5), cannabis use disorders (CUDIT), and severity of dependence (SDS) in all three groups (see Table 4; pre/post comparisons not reported). Assessment of the intervention satisfaction was completed by only a few participants at 6 months past baseline and we therefore omit group comparisons. Not surprisingly, those who remained in the active study arms rated their satisfaction as high (eg, intervention satisfaction in general [19/308, 6.2%]: very satisfied 42% [8/19], quite satisfied with most of the intervention 42% [8/19], quite unsatisfied with most of the intervention 11% [2/19], quite unsatisfied 5% [1/19]).

**Table 4:** Number of participants and mean and standard deviation changes from the imputed (50 imputations) and complete case datasets between baseline and 3-month follow-up.

Outcomes		Study arm 1 (self-help with chat) (n=114), mean (SD)		Study arm 2 (self-help without chat) (n=101), mean (SD)		Study arm 3 (waiting list) (n=93), mean (SD)	
		Baseline	Follow-up	Baseline	Follow-up	Baseline	Follow-up
**Frequency of cannabis use** ^a^							
	Imputed data	6.0 (1.6)	4.6 (2.1)	6.0 (1.6)	5.3 (1.8)	6.3 (1.0)	5.3 (1.8)
	Complete cases	6.1 (1.6)	3.8 (3.0)	6.1 (1.7)	5.5 (2.3)	6.7 (0.9)	5.3 (2.5)
**Quantity of cannabis use** ^b^							
	Imputed data	22.3 (14.8)	13.3 (12.0)	23.1 (23.1)	14.4 (11.8)	25.8 (18.7)	18.6 (17.7)
	Complete cases	23.0 (15.1)	10.9 (13.8)	25.1 (25.2)	14.2 (13.3)	23.6 (13.2)	20.7 (23.7)
**CUDIT** ^c^							
	Imputed data	19.8 (5.8)	16.6 (7.1)	19.7 (6.4)	15.6 (6.7)	19.1 (6.2)	16.6 (6.4)
	Complete cases	19.8 (5.8)	12.6 (8.4)	19.7 (6.4)	13.0 (7.4)	19.1 (6.2)	16.0 (7.2)
**SDS** ^d^							
	Imputed data	7.7 (3.5)	6.3 (3.3)	7.5 (3.6)	6.2 (3.1)	7.3 (3.1)	6.3 (3.3)
	Complete cases	7.7 (3.5)	5.3 (3.8)	7.5 (3.6)	6.0 (3.3)	7.3 (3.1)	5.9 (3.8)
**MHI-5** ^e^							
	Imputed data	53.9 (19.3)	58.1 (18.2)	53.9 (20.0)	60.4 (19.1)	55.1 (22.6)	59.4 (19.4)
	Complete cases	53.9 (19.3)	62.4 (19.8)	53.9 (20.0)	63.4 (20.4)	55.1 (22.6)	64.6 (18.3)
**Alcohol use in the last 30 days (risky)**							
	Imputed data	3.4 (6.2)	2.8 (2.8)	2.4 (5.0)	2.2 (3.0)	4.5 (7.9)	3.3 (4.0)
	Complete cases	3.4 (7.0)	1.6 (2.6)	2.5 (5.8)	1.0 (2.6)	4.5 (8.7)	2.1 (4.7)

^a^Based on the weekly number of cannabis use days according to the consumption diary.

^b^Based on the weekly number of standard cannabis joints according to the consumption diary.

^c^Cannabis Use Disorders Identification Test (CUDIT) scores range from 0 to 40 with a cutoff of >8 for a cannabis use disorder.

^d^Severity of Dependence Scale (SDS) scores range from 0 to 15 with a cutoff of ≥4 for cannabis dependence.

^e^Mental Health Inventory (MHI-5): higher values represent improved symptoms. MHI-5 values range from 0 to 100 with a cutoff of <70 for clinically relevant symptoms*.*

**Table 5:** Results for the between-study arm^a^ comparisons from the linear (and logistic) regression models and calculated effect sizes based on the imputed dataset (50 imputations).

Characteristics		beta	SE	*t*	*P*	Cohen's d (95% CI)
**Frequency of cannabis use** ^b^						
	(Intercept)	-3.95	0.58	-6.76	<.001	
	Arm 1 vs arm 3	0.70	0.32	2.16	*.03* ^*c*^	*0.20* (-0.07 to 0.47)
	Arm 2 vs arm 3	-0.05	0.33	-0.16	.87	-0.14 (-0.43 to 0.14)
	(Intercept)	-3.25	0.56	-5.79	<.001	
	Arm 2 vs arm 1	-0.75	0.32	-2.39	*.02*	*0.34* (0.07 to 0.61)
**Quantity of cannabis use** ^d^						
	(Intercept)	-14.50	2.24	-6.46	<.001	
	Arm 1 vs arm 3	4.73	2.50	1.89	*.06*	0.09 (-0.19 to 0.36)
	Arm 2 vs arm 3	3.77	2.42	1.56	.12	0.06 (-0.22 to 0.35)
	(Intercept)	-9.78	1.92	-5.09	<.001	
	Arm 2 vs arm 1	-0.96	2.43	-0.39	.69	0.01 (-0.26 to 0.28)
**CUDIT** ^e^						
	(Intercept)	-10.39	1.61	-6.46	<.001	
	Arm 1 vs arm 3	0.24	1.29	0.19	.85	0.09 (-0.18 to 0.37)
	Arm 2 vs arm 3	1.19	1.20	0.99	.32	0.21 (-0.07 to 0.49)
	(Intercept)	-10.14	1.68	-6.05	<.001	
	Arm 2 vs arm 1	0.95	1.16	0.82	.41	-0.12 (-0.39 to 0.14)
**SDS** ^f^						
	(Intercept)	-4.68	0.64	-7.34	<.001	
	Arm 1 vs arm 3	0.03	0.58	0.05	.96	0.08 (-0.19 to 0.36)
	Arm 2 vs arm 3	0.10	0.56	0.17	.86	0.07 (-0.21 to 0.35)
	(Intercept)	-4.65	0.65	-7.19	<.001	
	Arm 2 vs arm 1	0.07	0.55	0.13	.90	0.02 (-0.25 to 0.28)
**MHI-5** ^g^						
	(Intercept)	-43.91	4.33	-10.15	<.001	
	Arm 1 vs arm 3	0.96	3.44	0.28	.78	0.01 (-0.27 to 0.28)
	Arm 2 vs arm 3	-1.38	3.42	-0.40	.69	-0.09 (-0.38 to 0.19)
	(Intercept)	-42.95	4.14	-10.37	<.001	
	Arm 2 vs arm 1	-2.34	3.28	-0.71	.48	0.11 (-0.16 to 0.38)
**Alcohol use in the last 30 days (risky)**						
	(Intercept)	-2.84	0.56	-5.09	<.001	
	Arm 1 vs arm 3	0.32	0.63	0.52	.61	-0.10 (-0.38 to 0.17)
	Arm 2 vs arm 3	0.83	0.73	1.14	.25	-0.16 (-0.44 to 0.12)
	(Intercept)	-2.52	0.46	-5.46	<.001	
	Arm 2 vs arm 1	0.51	0.61	0.84	.40	0.06 (-0.20 to 0.33)

^a^Study arm 1: self-help with chat; study arm 2: self-help without chat; study arm 3: waiting list.

^b^Based on the weekly number of cannabis use days according to the consumption diary.

^c^Significant and borderline significant differences and effect sizes are in italics.

^d^Based on the weekly number of standard cannabis joints according to the consumption diary.

^e^Cannabis Use Disorders Identification Test (CUDIT) scores range from 0 to 40 with a cutoff of >8 for a cannabis use disorder.

^f^Severity of Dependence Scale (SDS) scores range from 0 to 15 with a cutoff of ≥4 for cannabis dependence.

^g^Mental Health Inventory (MHI-5): higher values represent improved symptoms. MHI-5 scores range from 0 to 100 with a cutoff of <70 for clinically relevant symptoms.

### Dropout Analysis

Dropouts at follow-up did not differ from completers with respect to the following baseline variables: gender (*t*=1.34, *P*=.16), age (*t*=-0.24, *P*=.81), years of cannabis use (*t*=0.18, *P*=.86), frequency of cannabis use in the preceding 30 days (*t*=0.22, *P*=.83), the weekly number of standardized cannabis joints used (*t*=1.20, *P*=.42), the SDS (*t*=-1.52, *P*=.13), the CUDIT (*t*=0.49, *P*=.63), alcohol use in the preceding 30 days (*t*=1.20, *P*=.23), risky alcohol use in the preceding 30 days (*t*=1.56, *P*=.12), and the MHI-5 (*t*=0.40, *P*=.69).

Significantly more participants could be followed up who received at least one chat session compared to those who could not be contacted at the 3-month follow-up (17.0% vs 5.5%, chi-square [df 2]= 7.5, *P*=.001).

Dropouts did not differ between the three study arms with respect to gender (*F*_2_ =0.04, *P*=.96), age (*F*_2_ = 1.13, *P*=.27), years of cannabis use (*F*_2_ = 0.81, *P*=.79), frequency of cannabis use in the preceding 30 days (*F*_2_ = 0.91, *P*=.59), the standardized cannabis use quantity (*F*_2_ = 0.93, *P*=.60), the SDS (*F*_2_ = 1.20, *P*=.23), the CUDIT (*F*_2_ = 0.94, *P*=.58), alcohol use in the preceding 30 days (*F*_2_ = 0.57, *P*=.97), risky alcohol use in the preceding 30 days (*F*_2_ = 0.48, *P*=.98), and the MHI-5 (*F*_2_ = 1.00, *P*=.47) at baseline.

### Nonintended Results

Although not intended as an outcome measure, we also offered cannabis abstention in the study protocol for those participants who wished to achieve this [1]. Self-reported 7-day point prevalence abstinence was significantly higher in self-help with chat (8.8%) than in the self-help without chat study arm (2.0%; beta = -1.56, SE = 0.79, *P*=.05, odds ratio [OR] = 0.21, 95% CI 0.02-2.33), but not between the self-help study arm with chat and the waiting list control group (4.3%; beta = 0.76, SE = 0.61, *P*=.21, OR = 2.14, 95% CI 0.86-5.30; see Tables 6 and 7).

**Table 6:** Number of participants in three study arms at each time point.

Study time point	Study arm 1 (self-help with chat) (n=114), n (%)	Study arm 2 (self-help without chat) (n=101), n (%)	Study arm 3 (waiting list) (n=93), n (%)
Week 1	9 (7.9)	12 (11.9)	N/A^a^
Week 6	8 (7.0)	9 (8.9)	N/A
Follow-up	9 (8.8)	2 (2.0)	4 (4)

^a^Not applicable (N/A).

**Table 7:** Self-reported abstinence between groups and with the corresponding logistic regression.

Abstinence at follow-up	beta	SE	*t*	*P*	OR^a^ (95% CI)
(Intercept)	0.04	0.02	1.88	.06	
Arm 1 vs arm 3	0.76	0.61	1.25	.21	2.14 (0.86-5.30)
Arm 2 vs arm 3	-0.80	0.88	-0.91	.36	0.45 (0.11-1.78)
(Intercept)	-2.34	0.33	-7.07	<.001	
Arm 2 vs arm 1	-1.56	0.79	-1.98	*.05* ^b^	0.21(0.02-2.33)

^a^Odds ratio (OR).

^b^Borderline significant difference is shown in italics.

### Subgroup Analyses

Participants in study arm 1 who received at least one chat session exhibited lower changes in their entries in the consumption diary. This meant that they took longer to complete the consumption diary and exhibited higher retention (change in mean 0.3 vs 0.5; beta = -0.28, SE = 0.12, *P*=.03, 95% CI -0.66 to -0.53) than those who did not receive the chat session in study arm 1. In line with this, they completed twice as many modules (mean 5.4, SD 2.8 vs mean 2.5, SD 2.1; *t*=5.45, df = 96, *P*<.001, 95% CI 1.88-4.00). Regarding cannabis use, these two subgroups did not differ in their reduction in frequency (change in mean 3.3 vs 1.9; beta = -1.38, SE = 0.93, *P*=.14, 95% CI -3.19 to 0.44) or quantity (change in mean 15.2 vs 10.6; beta = -4.61, SE = 4.44, *P*=.30, 95% CI -13.32 to 4.10).

Participants in study arm 1 who did not receive a chat session for whatever reason did reduce their frequency of cannabis use more (change in mean 1.9) than participants in study arm 2 (change in mean 0.7) who did not have the possibility for a chat session due to their allocation (beta = -1.97, SE = 0.60, *P*=.001, 95% CI -3.14 to -0.80). However, they did not differ with respect to the reduction in the quantity of cannabis used (change in mean 10.6 vs 10.3; beta = -0.33, SE = 6.48, *P*=.96, 95% CI -13.03 to 12.37). There were no significant differences between these two groups with respect to module completion (mean 2.5, SD 2.1 vs mean 2.9, SD 2.4; *t*=-1.18, df = 159, *P*=.23), but those in study arm 2 showed lower changes in their entries in the consumption diary (change in mean 0.4 vs 0.5; beta = -0.28, SE = 0.12, *P*=.03, 95% CI -0.66 to -0.53) compared to those who did not receive the chat session in study arm 1.

### Additional Help and Adverse Events

At the 3-month follow-up, 88.0% of participants (103/117) stated that they had not contacted any other treatment services (7 participants in study arm 1, 2 in study arm 2, and 5 in study arm 3). A total of 5.1% (6/117) had contacted a psychiatrist, 2.6% (3/117) a family doctor, 1.7% (2/117) a psychologist, 1.7% (2/117) a different Internet counseling service, and 1 person (0.9%) a drug counselor. During the whole study period, 5 out of 308 (1.6%) participants contacted one of the outpatient addiction clinics from the ARUD Centers for Addiction Medicine. None of them had to be treated as an emergency case or had to be referred to an inpatient treatment service. Moreover, none of the involved counselors or researchers are aware of any adverse or serious adverse event related to the Can Reduce study that was reported by other addiction counseling services.

## Discussion

### Principal Findings

The Can Reduce study could reach a different group of cannabis users who do not enter outpatient addiction treatment services. They are older and consume much more cannabis than outpatient service users. The finding that we reached cannabis users with more entrenched problems (eg, daily users) is not consistent with the common perception that those using online interventions have less severe problems than those entering outpatient services. We assume that this finding was most probably due to an age effect. Older users consume longer and possibly also more than younger ones but might feel more stigmatized if they enter an outpatient addiction service, due to their greater responsibilities and roles in social relationships, at work, and in society in general.

Can Reduce participants allocated to the self-help with chat study arm reduced their frequency of cannabis use more than those in the other two arms. Even cannabis abstinence was higher among those who received additional chat counseling relative to those who received self-help only at follow-up. There was a trend (*P*=.06) for a greater reduction in quantity of cannabis use in those who received chat versus those in the waiting list group and only a weak tendency (*P*=.12) for the comparison of those with self-help only versus waiting list. Hence, adding one to two chat counseling sessions that are tailored to the self-help participant data and are based on the same therapy approaches as the self-help part can be worthwhile.

As only one-quarter received at least one chat session, the question arose as to what was actually responsible for the superiority of the self-help with chat study arm. The subgroup analyses showed that those participants in study arm 1 who did not receive a chat session reduced their frequency of cannabis use more than those who received self-help only from the beginning (study arm 2). Thus, even an invitation to a chat session and the knowledge that there is a possibility to have a chat appointment might have improved this main outcome for cannabis use. To the best of our knowledge, there are no similar studies in the literature that have reported a comparable effect. However, our result is in line with the first point of the Supportive Accountability model [39] that argues that human support increases adherence—and potentially outcomes—through accountability to a coach who is seen as trustworthy, benevolent, and having expertise. We took care that our chat counselors were perceived as possessing these attributes in the respective chat study arm.

However, those participants who actually received at least one chat counseling session in study arm 1 still performed better in their reduction of cannabis use and completed more self-help modules than their counterparts who did not receive a chat session in the same study arm. This result is in line with a further point of the Supportive Accountability model [39] expecting better outcomes due to a reciprocal relationship, through which the patient can derive explicit benefits. However, this finding could also be related to a selection bias. Those who actually received at least one chat appointment with their counselor could be a selected group of more compliant and possibly more structured participants who could profit best from their allocated intervention.

If we compare the current results with former studies about the reduction of cannabis use with similar therapeutic approaches, it stands out that participants in the Can Reduce self-help without chat study arm performed worse than those in the Australian *Reduce Your Use* study [15], in which greater effects were achieved in the reduction of the quantity (d=0.06 vs d=0.25 in the Australian sample) and frequency of cannabis use days (d=-0.14 vs d=0.33). This Australian study enrolled cannabis users of a similar age range, but included more females (38.6% vs 24.7%) and users with less severe cannabis consumption at baseline. This may also be the reason that we did not observe greater effects in the Severity of Dependence Scale, in contrast to the Australian study (ITT: d=0.07 vs d=0.33). However, the Australian study provided videos of a real person who provided continuous MI during almost all parts of the intervention. This clearly might have been an advantage compared to our version with only written MI. Another possibility that could potentially increase the engagement of self-help participants might be to provide a personal companion with whom the participants could identify, as we attempted in a similar ongoing trial with problematic cocaine users [40]. The effects in the Can Reduce self-help plus chat study arm were smaller for the quantity of cannabis used (ITT: d=0.09 vs d=0.25) and similar for the frequency of cannabis use days (ITT: d=0.34 vs d=0.33) compared to the Australian self-help trial [15]. Participants in the Can Reduce self-help with chat study arm performed better than those in the more recent German *Quit the Shit* study with respect to the reduction in the frequency of cannabis use days (ITT: d=0.34 vs d=0.20) [11]. The German study recruited younger participants (mean age 24.2 years, SD 5.8 vs mean age 29.8 years, SD 10.0).

We observed a borderline significant effect in the abstention rates between the self-help with chat and the self-help without chat study arms. As we did not initially expect that enough participants would maintain their abstinence, we omitted abstinence as an outcome measure in the study protocol [1]. Abstinence rates were not reported in the German *Quit the Shit* studies [12,14], but comparable differences between study arms with respect to 3 months of abstinence were achieved in this study (8.8%) and in the Australian study (5.8%) [15].

Setting a goal for cannabis consumption was implemented as described in the study protocol [1]. In the introduction to the consumption diary, we recommended that participants should plan to reduce their cannabis use by at least 20 to 30% in the first week and then continue with this strategy in subsequent weeks if they succeeded. For participants who did not succeed, we recommended that they created more modest goals until their final aim was achieved. During the analyses of the consumption diary patterns, we realized that there was a considerable subgroup of participants who preferred to abstain from cannabis even in the first week. Experiences from the chat counseling sessions showed that, although the counselors in the corresponding study arm strengthened this procedure in the self-help intervention part, some participants argued that they had learned from previous experience that they were much more successful in stopping a potentially addictive behavior than in reducing it. In this case, the counselors tried to encourage them to abstain and to assist them in the maintenance of their abstinence. However, this also resulted in some cases with a new challenge. There was a substantial number of participants in this subgroup who very quickly abstained from their cannabis use and who did not log in again, although they were reminded by automated reminder emails and/or their chat counselor, and who then could not be reached at the follow-up assessment. Possible strategies to prevent such early missing cases due to abstention could be specific reminder emails sent automatically and/or by introducing the chat counselor at an earlier stage.

### Strengths and Limitations

The strengths of the Can Reduce study are that the intervention is theory based and pretested, that this Web-based intervention was able to reach cannabis users who otherwise would not have sought help, and that we were able to disentangle the effects of chat counseling additional to self-help for the reduction in cannabis use in frequent cannabis users, three-quarters of whom used cannabis daily. This study also possesses limitations that merit consideration. First, we did not biologically validate cannabis consumption for financial reasons, as we did not want to limit participation to participants who were willing to provide, for example, saliva samples, and as we did not want to limit external validity. Second, we did not succeed in attaining a better 6-week follow-up as intended in the study protocol, which limits the explanatory power of the short-term effects of Can Reduce. However, the 3-month follow-up rate (117/308, 38.0%) was comparable to similar studies with problematic cannabis users in Europe [12,14], but rather low compared to Internet-based randomized controlled trials for the improvement of nonaddiction-related problems. Moreover, we used the most reliable imputation method available to handle missing data [38] at follow-up. Third, due to ethical legislations, we had to limit the minimal participation age to 18 years, as younger participants would have needed parental informed consent, and we expected that the overwhelming majority of minors would avoid participation under these conditions. Moreover, this would have been a contradiction with the concept of a maximally anonymous Web-based intervention for the reduction of cannabis use. Cannabis is still illegal in Switzerland and Germany, from where the majority of participants in this study come from. Fourth, participants were randomly allocated into three study arms with slightly different sizes and a block randomization could have prevented this.

### Conclusions

In conclusion, the Can Reduce study demonstrated that Web-based interventions possess the potential to reach heavy cannabis users who differ from those who enter outpatient addiction treatment services. We further conclude that offering brief chat counseling in addition to Web-based self-help can significantly increase success in the reduction of cannabis use in the different groups of cannabis users investigated*.*

## Appendix

Multimedia Appendix 1 Can Reduce participant information and informed consent page.

Multimedia Appendix 2 CONSORT-EHEALTH checklist V1.6.1 [28].

## Abbreviations

act-info: addiction, care and therapy information
ANOVA: analysis of variance
ARUD: Arud Centers for Addiction Medicine
BSM: behavioral self-management
CBT: cognitive behavioral therapy
CCS-7: Cannabis Craving Symptoms questionnaire
CUDIT: Cannabis Use Disorders Identification Test
CWS: Cannabis Withdrawal Scale
FDA: Fragebogen Substanzanamnese (questionnaire for the assessment of substance use history)
ISGF: Swiss Research Institute for Public Health and Addiction
ITT: intention-to-treat
MHI-5: Mental Health Inventory
MI: motivational interviewing
N/A: not applicable
OR: odds ratio
RCT: randomized controlled trial
SDS: Severity of Dependence Scale
WL: waiting list
