# Supplementary material for: A Web-Based Self-Help Intervention With and Without Chat Counseling to Reduce Cannabis Use in Problematic Cannabis Users: Three-Arm Randomized Controlled Trial
Source: J Med Internet Res. 2015 Oct 13;17(10):e232. doi: 10.2196/jmir.4860 (PMC4642392; doi:10.2196/jmir.4860)
Supplement: Multimedia Appendix 1 [file jmir_v17i10e232_app1.pdf]

Teilnehmerinformationen und Einverständniserklärung

Sehr geehrter Teilnehmer, sehr geehrte Teilnehmerin,

Es freut uns, dass Sie sich für die Teilnahme an unserer Studie interessieren. Bitte nehmen Sie sich einen Augenblick Zeit und lesen Sie den folgenden Text aufmerksam durch.

Welches Ziel hat diese Studie?
Ziel der geplanten Studie ist es, die Wirksamkeit einer Internethilfe zur Reduktion von Cannabiskonsum bei Cannabiskonsumentinnen und -konsumenten zu testen. Bisher existiert eine Internethilfe aus Australien, welche sich in einer ähnlichen Studie wie wir sie hier durchführen als wirksam erwies. Wir wollen testen, ob dies auch hierzulande klappt.
Dazu sollen Cannabiskonsumentinnen und -konsumenten vor, während und drei Monate nach ihrer ca. sechswöchigen Benützung der Selbsthilfeoberfläche mittels standardisierten Online-Fragebögen befragt werden, die ihren Cannabiskonsum reduzieren oder ganz damit aufhören möchten.

Ihre Beteiligung an der Studie
Sie können bei dieser Studie teilnehmen, so fern Sie genügend Deutsch verstehen und lesen können, Zugang zum Internet haben und sich mit der Benützung von herkömmlichen Internetseiten auskennen. Weiter wird vorausgesetzt, dass sie aktuell keine anderweitige Behandlung zur Reduktion des Cannabiskonsums beanspruchen und bei weiblichen Teilnehmerinnen, dass Sie nicht schwanger oder am Stillen sind.
Da die Studie von der Schweizerische Koordinations- und Fachstelle Sucht finanziell unterstützt wird, ist Ihre Teilnahme kostenlos.
Bitte beachten Sie, dass die Teilnahme bei dieser Studie nicht eine Behandlung von Cannabisabhängigkeit durch eine fachärztliche Person ersetzen kann. Adressangaben zu solchen Fachpersonen finden Sie auf der Studienwebseite. Zudem wird Ihnen beim Auftreten von akuten Entzugssymptomen oder anderen gesundheitlichen Problemen während und nach der Studie geraten eine fachärztliche Person unabhängig von der Studie aufzusuchen.

Freiwilligkeit
Die Teilnahme an dieser Studie ist freiwillig. Ihr Einverständnis kann jederzeit und ohne Angabe von Gründen widerrufen werden.

Schweigepflicht-/Datenschutz
Alle Personen die an der Durchführung dieser Studie beteiligt sind unterstehen der Schweigepflicht und sind dem Datenschutzgeheimnis verpflichtet. Die Daten werden in anonymisierter Form behandelt, das heisst ohne Namen und Adressen. Einzig eine E-Mail Adresse sowie eine Telefonnummer zur Wiedererreichbarkeit bei der Nachbefragung nach 3 Monaten werden zu diesem einen Zweck für die Studienteilnahme verlangt. Zu beidem Fragen wie Sie erfolgter Registrierung bei Cannabis Control. Die Einwilligungserklärungen verbleiben unter Verschluss im Schweizer Institut für Sucht- und Gesundheitsforschung für den Zeitraum der gesetzlichen Aufbewahrungsfrist und werden danach automatisch vernichtet.

Falls Sie noch Fragen haben, dann wenden Sie sich bitte direkt an folgende E-Mail Adresse: cannabiscontrol@isgf.uzh.ch.

Einwilligung zur Studienteilnahme

Bitte lesen Sie die zur Studienteilnahme notwenigen Punkte zur Einwilligungserklärung aufmerksam durch. Wenn Sie damit einverstanden sind, dann bestätigen Sie dies durch Ankreuzen der dafür vorgesehenen Kästchen und schicken Sie das vorliegende Formular durch Mausklick auf die untenstehende Taste „Formular abschicken" ab. Sobald sie dies getan haben, werden Sie unter Anleitung dazu aufgefordert ein anonymes Login und Passwort zu erzeugen, sind bei Cannabis Control als User registriert und haben jederzeit Zugang zu Cannabis Control.

☐ Ich erkläre hiermit, die Teilnehmerinformationen zur wissenschaftlichen Untersuchung und die Einwilligungserklärung erhalten zu haben.

☐ Ich bestätige, dass ich die schriftliche Teilnehmerinformationen gelesen und verstanden habe, ich freiwillig an der Studie teilnehme und ich meine Teilnahme jederzeit und ohne Angaben von Gründen beenden kann.

☐ Ich bestätige, dass ich damit einverstanden bin, dass die im Rahmen dieser Studie erhobenen Daten vollständig anonymisiert ausgewertet werden: Es wird gewährleistet, dass sämtliche personenbezogenen Daten (z.B. Alter, Geschlecht) nicht an Dritte weiter geben werden. Meine persönlichen Daten unterliegen dem Datenschutz. Die Publikation der Studienresultate wird Angaben über die Gesamtpopulation der Teilnehmer beinhalten, aber keine Daten einzelner Personen.

Wenn Sie wünschen, dass Sie über die Gesamtresultate dieser Studie informiert werden, dann kreuzen Sie dies bitte hier an. Wenn Sie das nicht wünschen, dann lassen Sie dieses Feld einfach leer.

☐ Bitte informieren Sie mich über die Resultate der Studie.

Bitte retournieren Sie Ihre Einwilligung zur Studienteilnahme direkt via dem vorliegenden Formular über unsere Webseite, als ausgedruckte Word Datei an folgende E-Mail Adresse: cannabiscontrol@isgf.uzh.ch

oder ausgedruckt auf dem Postweg an folgende Adresse:

Schweizer Institut für Sucht- und Gesundheitsforschung ISGF
Cannabis Control
Konradstrasse 32
Postfach
8031 Zürich

Formular abschicken
